# PEER REVIEW PROCESS OF CAREER DEVELOPMENT AWARDS (K) APPLICATIONS AT NIA

**DOI:** 10.1093/geroni/igad104.0663

**Published:** 2023-12-21

**Authors:** Ramona Gianina Dumitrescu, Bita Nakhai

**Affiliations:** National Institute on Aging, Bethesda, Maryland, United States; National Institute on Aging NIA), National Institute of Health (NIH), Bethesda, Maryland, United States

## Abstract

In this presentation, Dr. Dumitrescu will take you behind the scenes of what occurs in a peer review meeting of career development award (K) applications at NIA. Some of the mechanics of the peer review process will be discussed, including composition of study groups, general timelines, and how the results of the peer review process are communicated to applicants via Summary Statements. Attendees will understand how reviewers evaluate the 5 core domains that generate a final impact score, including the: 1) candidate’s qualifications; 2) the career development plan and career goals & objectives; 3) research plan; 4) mentor(s), collaborators & consultants; and 5) institutional environment & commitment to the candidate. Dr. Dumitrescu will help to de-mystify the peer review process in order help applicants leverage their respective applications for maximum success.

